# Harvard Odontological Society

**Published:** 1892-12

**Authors:** 

**Affiliations:** Harvard Odontological Society


### HARVARD ODONTOLOGICAL SOCIETY.

A REGULAR monthly meeting of the Harvard Odontological Society was held on Thursday evening, September 29, 1892, at Young's Hotel, Boston, Dr. Werner being in the chair.

The paper for the evening was read by Edwin C. Blaisdell, D.M.D., of Portsmouth, N. H.; subject, "Operative Failures and their Remedies."

For Dr. Blaisdell's paper, see page 868.

### DISCUSSION.

*Dr. Smith.*—Mr. President and gentlemen: I am particularly pleased to-night to hear this paper, because it became my duty not long ago, as one of the members of the Faculty, to consider the fitness of certain men as instructors in the Dental School of Harvard University, and it was my pleasure to consider the name of Dr. Blaisdell, and to favor his appointment; and after hearing his paper this evening I am very glad that I did, and feel that the school has been benefited by his selection as one of the instructors.

Following the line of his arguments, I can simply say that in the main I agree with and endorse what he has said on the points mentioned. I know it is claimed by some that gutta-percha at the cervical wall in approximal cavities is not at all necessary; that a cement properly mixed and properly placed in the cavity would stand equally well at the cervical margin as gutta-percha. That has not been my experience, even when I have used the matrix; and so, notwithstanding the fact that this opinion seems to be held by some competent men, I consider that for the general practitioner it is always much safer to use gutta-percha.

The essayist spoke of the danger arising from the use of gutta-percha fillings in crowns with frail walls. I have never used gutta-percha as a crown filling, but I have seen the same result that he speaks of,—the enamel being fractured, and, as he says, in such places that it was very evident that the fracture was caused by the expansion of the gutta-percha without the aid of masticating power.

Referring to the use of copper amalgam, I have never had any experience in it at all, as I have never used it. We heard a great deal about it at one time, and Dr. Rollins, who experimented with it, was quite enthusiastic and wrote some papers recommending its use ; but he has since taken it all back, so I consider that I have not lost anything in waiting for somebody else to experiment and find out whether it was of value or not.

The question as to whether we should use amalgam as a filling, is one which I should like to have settled scientifically. There are many cases where we put in amalgam fillings, and they seem to be doing good service until some day the patient becomes ill, and the physician proclaims that you are poisoning the patient; for that reason we have to be careful in its use, and I shall be glad when the time comes that we have a scientific solution of the matter, and have it definitely settled one way or the other.

The use of gold and amalgam in combination is a method of filling that I have employed for some time, and have fillings that have been in a great many years which are still in excellent condition, and I have never yet seen cause for regret in its use. I don't remember when I first began to use it, but I was led to it first by patching gold fillings with amalgam. We often see a beautiful gold filling extending from the cervical wall and including part of the crown ; that filling has given out at the cervical wall. It may be one of our own, or it may be the work of an operator who has the reputation of being very skilful ; and when it is, we are rather apt to be glad than otherwise, for we know so well that ours fail in the same way in spite of all our care ; we have patched those fillings with amalgam and found that they have preserved the teeth. In this way I began many years ago to use amalgam in the commencement of this kind of fillings, and then at the next sitting introducing gold ; but I think it is to Dr. Clapp that we owe the combination filling at one sitting. I cannot say that he invented it, but he has certainly impressed it upon our minds, and Dr. Clapp's name will be associated with that method of filling because of his efforts to promote its general adoption.

The method of shoeing teeth is a good one. I have never used it in a live tooth, but I have practically with pulpless teeth, having the pin go into the root in a similar way. Long ago, when a student with my preceptor, Dr. Shaw, a number of teeth were shod by him in that way. We often read of something supposed to be new which we practised several years before. In looking over some old teeth recently I found some shod in this way; but it is an excellent way of treating teeth, nevertheless.

It struck me when I read the subject, "Operative Failures and their Remedies," that some were not due to the lack of skill or to the lack of method, but in many cases to a lack of time,—we are apt to operate too hurriedly. Many dentists charge too small a fee; their business increases, and it is money in pocket to see as many patients in a day as possible. When that time comes, that moment their skill as an operator declines. There are a great many young dentists who have failed as operators because they have tried to see too many patients. Now, gentlemen, every Harvard graduate—at least all that I know anything about—is skilful enough to charge more than the average fee for the service rendered, and it behooves us all for our health and for our reputation to bear this fact in mind. Take time enough to do your work thoroughly, charge a respectable and just fee, and one great cause of operative failures will be removed.

*Dr. Cooke.*—I have seen some cases where copper amalgam has been very successful, and has preserved the teeth where nothing else under the circumstances would; but I have seen a great many more cases, especially in approximal fillings, where it has dissolved exactly as an oxyphosphate filling does; for that reason, and for others given in my paper last spring, I have used this material but a very little during the past year, and then only where I felt obliged to, in repairing fillings.

While this method of shoeing teeth would be very good in some cases, a great many teeth are not strong enough at the edges for such treatment. Two holes in that situation would certainly weaken it, especially if it had an approximal cavity.

I agree with what Dr. Smith has said about hasty operations which result from the dentist charging too small a fee and being too busy. It is not a question in dentistry of what a man can do, but of what he does do,—it depends not so much on his ability, as upon his conscience. Many men can do good work, but become careless, excavate only slightly, and the patient finds subsequently that the work was not properly done. I am firmly convinced that the decaying material should all be cut out. We have repeatedly seen decay left in cavities and covered with gold; in such cases you know the cavity has not been properly excavated.

*Dr. Page.*—I am very much encouraged by what Dr. Smith said about charging respectable fees and operating for fewer patients. I do not believe in working so many hours a day as some do, but I do believe in using natural forces and saving our own energy, which will assist you to find time to overcome more failures.

*Dr. Upham.*—I want to speak, in this connection, about the cavities at the cervical wall in bicuspids and molars, and, in fact, all teeth excavated with a convex instrument like a spoon excavator, which leaves a sharp edge or acute angle at that point instead of an obtuse angle or bevelled edge. Some instrument should be used which makes a bevelled edge, leaving an obtuse angle at least upon the enamel rods, when the cavity is finished. I will show some instruments that I use for that purpose. They explain themselves. The failure of many approximal fillings is due to improper excavation here, instead of using wrong materials. A spoon excavator in approximal cavities where teeth are very close together cannot be used on the margin of the cavity so as to give even a right angle. There will be a convex cut there, leaving a sharp or acute-angle edge. There should always be at the cervical wall a bevelled edge,—the enamel bevelled surely, to avoid fracture in condensing gold.

*Dr. Grant.*—What Dr. Blaisdell says about oxyphosphate fillings I endorse most thoroughly, and although it is the universal practice among men who try to get the best results in such fillings to use gutta-percha, I think in many instances gutta-percha is very poor material to use. If the tooth is sensitive, it will not bear the pressure of a filling at the cervical wall. I don't know just what to use. Of course, I feel certain that the cement will dissolve, but I do it because I can't do better. In some instances I have used amalgam and put oxyphosphate over it, and it is generally far enough under the gum to be so that it is beyond the reach of thermal changes, and works very well. At other times I simply use oxyphosphate and expect it to dissolve.

I do not have the very best success always in working with a matrix. I often find teeth very small at the neck and large at the grinding surface, and it is laborious to cut down the surface made under the matrix, which is almost a perpendicular line; of late I have been using a hand instrument. It is simply filed down thin as a steel matrix, and, holding that in one hand, I can fill with gutta-percha, or even with amalgam, and contour the tooth, and do not have so much to do at the close.

I have used the gold and amalgam as Dr. Smith described, which is to fill partly up with amalgam and allow it to set, and at the next sitting introduce the gold. It is a good way of making large fillings,—far better than making them all of gold.

I am an advocate of soft gold at the cervical margin. I do not believe that cohesive fillings are adapted there. I should never feel, when putting one in, that it was permanent.

*Dr. Perrin.*—The matrix has been spoken of, and I am sure I have less failures when I use it. I have tried to fill cavities at the cervical walls without it, and find I can work them much better with it. At the cervical margin of proximal cavities in bicuspids and molars I consider a matrix indispensable.

*Dr. Blaisdell.*—I would like to ask Dr. Perrin if he holds his matrix in place rigidly, or loosely, in order to get that little bulge?

*Dr. Perrin.*—I like to have them fit closely. I use a matrix that is applied very easily; I believe the name of it is the Ladmore-Brunton. I always use the crystal gold, or Steurer's gold, to start my filling, and pack against the matrix.

*Dr. Hopkins.*—I never saw a matrix that would not bulge in packing the gold sufficiently to contour.

*Dr. Shepherd.*—Excluding moisture is very important. I use the rubber dam a great deal more now than formerly. We often try to fill small, inaccessible cavities hurriedly, using a napkin and putting in amalgam; and as we do not want the amalgam to set, are apt to be careless about having the cavity perfectly dry, and secondary decay is almost sure to follow.

As far as undercuts go, I think the smaller the undercut the better. In many cases I make no undercut. I merely have the inside of the cavity a little larger than the outside, and get rid of the angles in that way.

HENRY L. UPHAM, D.M.D.,
*Editor Harvard Odontological Society.*

